# Exploration of Spontaneous Coronary Artery Dissection: Pathophysiology, Diagnosis, Management, and Clinical Implications

**DOI:** 10.31083/RCM45172

**Published:** 2025-12-22

**Authors:** Aro Daniela Arockiam, Praveen Bharath Saravanan, Priyansha Singh, Aonghus J. Feeney, Ankit Agrawal

**Affiliations:** ^1^Internal Medicine, Unity Health-White County Medical Center, Searcy, AR 72143, USA; ^2^Internal Medicine, KAP Viswanatham Government Medical College, TN 620001 Tiruchirappalli, India; ^3^Internal Medicine, Smt. Nathiba Hargovandas Lakhmichand Municipal Medical College, GJ 380006 Ahmedabad, India; ^4^School of Medicine, College of Medicine, Nursing and Health Sciences, University of Galway, H91 TK33 Galway, Ireland; ^5^Cardiovascular Medicine, University of Arkansas for Medical Sciences, Little Rock, AR 72205, USA

**Keywords:** spontaneous coronary artery dissection, atherosclerosis, coronary artery disease

## Abstract

Evidence is accumulating that shows spontaneous coronary artery dissection (SCAD) as a recognized cause of acute coronary syndrome (ACS), disproportionately affecting younger people and women. Moreover, despite continuing progress, the understanding of the pathophysiology, diagnosis, and management of SCAD remains limited. SCAD, by definition, is a non-atherosclerotic formation of an intramural hematoma or intimal tear, yet current diagnostic criteria and management are derived from atherosclerotic ACS guidelines. This review encompasses the current understanding of the condition, including risk factors, diagnostic and imaging modalities available for detection, differentials to be considered, associations with other comorbidities, prognostic factors, and management options for both the short and long term, encompassing both medical and interventional therapies. Meanwhile, a lack of research in key populations, such as non-pregnant women, postmenopausal women, and men, prevents the generalizability of these findings and has been highlighted. However, by identifying and conceptualizing existing evidence, this review aims to provide direction to future research.

## 1. Introduction

Acute coronary syndrome (ACS), myocardial infarction, and sudden cardiac death in young women and individuals with few conventional coronary artery disease (CAD) risk factors are now being recognized as due, in part, to spontaneous coronary artery dissection (SCAD) [1]. The formation of an intramural hematoma (IMH) or intimal tear within the coronary wall can lead to inadequate blood flow and subsequent ischemia or infarction of the myocardium. SCAD is a rare entity, yet pathologists found it accounted for up to 4% of acute myocardial infarction cases among those studied [2]. Current research indicates it also accounts for 35% of sudden cardiac deaths among women under 50 years [3]. Unrelated to atherosclerosis, SCAD may have a genetic basis. The condition may also be stress-related, and some suggest SCAD may relate more closely to dissecting aortic aneurysm than to atherosclerosis. Dissection of the coronary artery presents unique diagnostic and therapeutic challenges, and support is needed for those diagnosed to move from life-threatening events to healthier states [4].

This review intends to furnish a complete picture of SCAD, covering such aspects as its epidemiology, pathophysiology, and clinical presentation. We discuss recent advancements in the field and ongoing controversies to synthesize a coherent account of SCAD. From that account, we derive some knowledge gaps that could be explored in future research to improve individual outcomes in this complex and multifaceted condition.

## 2. Review

### 2.1 Epidemiology

It is difficult to predict the precise global incidence of SCAD due to underdiagnosis, limited physician awareness, and inconsistent availability of diagnostic resources, especially in low- and middle-income regions [2]. Ninety percent of SCAD patients are women aged 47–53 years, with SCAD accounting for 21%–27% of pregnancy-related myocardial infarction [5, 6]. The incidence is likely underestimated due to a low index of suspicion in young women without traditional cardiac risk factors [7]. Pender *et al*. [8] cited SACD registries such as the Canadian (n = 1173, 89.5% female) and Australian-New Zealand (n = 505, 88.7% female), which further confirms the gender-specific prevalence. Males account for 10% of such dissection cases [9]. Fahmy *et al*. [10] reported that in men, major precipitating factors include isometric exertion and, notably, fibromuscular dysplasia, which was observed in half of these cases. This was significantly higher than in the general male population [10]. Würdinger *et al*. [11] have documented an increasing trend in SCAD, not due to an increased incidence of disease in the population but attributed to improvement in clinician awareness and better knowledge of its angiographic appearance. Therefore, there are several factors that warrant special attention to assess the likelihood of SCAD, including gender, age, genetic background, clinical presentation, presence of triggering factors, hormonal changes, etc. [12].

### 2.2 Adolescents and Children

SCAD, as stated, is more common in women around 40–50 years [13]. The occurrence of SCAD among adolescents is less reported [14]. The existing handful of case reports indicate possible triggers as caffeinated “energy drinks”, methylphenidate, heavy exercise, and associations with diseases including systemic lupus erythematosus and neurofibromatosis [15, 16, 17, 18, 19, 20]. They have presented either asymptomatically or with chest pain and have a male preponderance for a small sample size. One case reports a presentation of a 16-year-old with syncope causally associated with heavy exercise [14]. To date, no guidelines exist for the evaluation and management of SCAD in adolescents, nor for the long-term risks and management strategies following the event. Registries and studies reporting the data of SCAD among younger people are scarce and required in the future.

### 2.3 Pathophysiology

Pathophysiological mechanisms of SCAD are unrecognized but presume structural instability of the arterial wall. Inflammation of the vessel wall occurs from conditions including fibromuscular dysplasia (FMD), connective tissue diseases including Marfan syndrome, Loeys–Dietz syndrome (LDS) and Ehlers–Danlos syndrome (EDS), and peripartum hormonal fluctuations [2, 21]. It can start because of extreme emotional or physical stress. The dissection makes a simulacrum in which blood pools, reducing the real hole and decreasing blood flow [4]. The result of this ischemia is ACS, from unstable angina to myocardial infarction. Women are most vulnerable and often have no known risk factors for atherosclerosis. Genetic studies have found a higher than expected frequencies of genes attributed to SCAD among populations with FMD and LDS, thus inviting the screening of FMD, LDS, and EDS among SCAD patients [2, 22, 23]. Such genes include pathogenic *PKD1*, *FLNA*, *LOX*, and *SMAD3* variants, but the chances of these occurrences creating a SCAD event are still <5%, and hence, SCAD is not considered a familial trait [2, 3, 21]. More conclusive genetic studies are imperative.

### 2.4 Environmental Risks

The triggers of a SCAD event are multifactorial, primarily involving genetic predispositions, hormonal factors, and vascular conditions, which are further influenced by environmental stressors and precipitants [24]. In a cohort reported in Fahmy *et al*. [10], isometric exertion was found to be the dominant precipitating factor in 44% of men, of whom the majority were involved in lifting heavy weights. Additionally, emotional stressors were a significant contributor to the incidence of SCAD in women. Such findings have been corroborated in another study by Daoulah *et al*. [25], across four Arab Gulf countries. Among the various emotional factors contributing to SCAD, unemployment is associated with high reported levels of emotional stress among individuals seeking employment [26]. According to a study done from the genetic spontaneous coronary artery dissection (GSCAD) Registry [27], unemployed patients with SCAD experienced significantly worse adverse cardiovascular events. Studies have attributed this effect to catecholamine surges, which increase arterial shear stress through enhanced myocardial contractility or vasospasm, ultimately predisposing to intimal rupture of the vasa vasorum [28]. Although rare, specific environmental factors have been associated with the incidence of SCAD. One such case was highlighted by Mahendiran *et al*. [29], where the first case of SCAD caused by scuba diving was documented. While catecholamine surge was the contributory mechanism, it was further complemented by various diving factors that needed to be taken into consideration for the incidence of SCAD. Exposure to specific stressors such as hydrostatic pressure, hyperoxia-induced vasoconstriction, and elevated cardiac filling pressures could have further amplified the sympathetic nervous system activity [30, 31]. Implementation of the Valsalva maneuver for vigorous ear pressure equilibrium could serve as another contributing factor as well [29]. Skiing was another trigger that was documented in a case report where a 48-year-old Caucasian developed a ripping chest pain while skiing off-piste at an altitude of more than 3300 m [32]. The adverse cardiovascular event could be attributed to the reduced availability of oxygen and the associated environmental factors (exercise, dehydration, thermal stress, and emotional stress from personal danger) [33]. To better understand the relationship between environmental stressors and SCAD, larger and long-term prospective studies are required.

## 3. Types of SCAD

Saw [34] proposed distinctive angiographic types of SCAD to suggest further imaging modalities to confirm or disprove the diagnosis and to indicate treatment.

SCAD type 1 [34] is present in about 10%–15% of cases, where it has an angiographic appearance of an arterial dissection due to the intimal tear spreading longitudinally. It illustrates the characteristic appearance of arterial wall contrast staining, showcasing multiple radiolucent lumens, which may or may not exhibit dye retention or a delayed clearing of contrast. Intravascular ultrasound (IVUS) (optical coherence tomography (OCT) preferred) is used for confirming the diagnosis and guiding the true lumen wire placement to give optimized results. Being the most prevalent, occurring in 60–70% of patients, SCAD Type 2 [34] has features of diffuse smooth stenoses that vary in severity and length, often exceeding 20 mm. This type typically presents with sudden changes in arterial caliber from the normal diameter to diffuse smooth narrowing. While type-2A lesions will present with normal caliber, both proximal and distal to the dissection, the type-2B variant is diagnosed when the lesion extends up to the terminal end of the vessel, with complete distal vessel involvement. Here, the intravascular imaging is reserved only for ambiguous cases, as conservative treatment is recommended in the majority. Type 3 [34] pertains to a focal or tubular stenosis, usually less than 20 mm in length, which resembles atherosclerosis and requires additional diagnostic techniques to make a precise diagnosis. As SCAD-related total vessel occlusion could not fit into the classification, Al-Hussaini and Adlam [35] proposed SCAD type 4, which stated a total vessel occlusion after the exclusion of coronary embolism. Additionally, following the natural history of SCAD, it should display healing of the complete vessel on subsequent angiography [36].

## 4. Diagnostic Criteria and Modalities

Accurate and timely diagnosis is critical, as management differs significantly from typical ACS, usually favoring conservative approaches rather than interventional strategies [37]. Assessment of the incidence should include thorough history taking and physical examination, especially if the patient belongs to a high-risk group [7]. Diagnostic modalities that are being adopted to differentiate SCAD from other causes of ACS include invasive coronary angiography (ICA), IVUS, and OCT.

### 4.1 Coronary Angiography 

During an emergency setting, it is difficult to distinguish SCAD from a typical atherosclerotic myocardial infarction; therefore, intracoronary angiography is considered the predominant first-line imaging modality due to its universal availability [38]. Retrospective studies have reported that type 2 is the most common, followed by type 1 and type 3, with the left anterior descending artery being the most frequently affected [39]. The major disadvantage of coronary angiography is that the 2-dimensional luminogram limits the assessment of the pathological process that is affecting the coronary wall [40]. Thus, supplementary imaging methods such as OCT and IVUS are further adopted to confirm the diagnosis and provide visualization of the vessel wall tear.

### 4.2 Intravascular/Intracoronary Imaging Modalities

The characteristics of SCAD-type lesions, such as the existence of an intimal flap and the presence and length of extension of intramural hematoma, along with the presence of a possible thrombus, can be well detailed by OCT and IVUS [2, 40].

Maehara *et al*. [41] reported the role of IVUS in the diagnosis of SCAD in patients without characteristic angiographic features. With an axial resolution of 150 µm, IVUS can differentiate the two lumens while reporting the severity of false lumen thrombosis [37, 41, 42]. As this modality has a greater vessel wall penetration [42, 43], it offers better evaluation of the extent of intramural thrombus, with deeper vessel visualization. OCT is preferred to IVUS due to higher image resolution, which results in better identification of intimal tears and flaps, along with confirming the guidewire position in the true lumen [42, 43]. However, the cost of high-resolution images in OCT comes with a major disadvantage of potential hydraulic propagation of the dissected segment due to the requirement of blood clearance for contrast medium injection [44].

The use of intracoronary modalities may present complications of extension of coronary dissection with guidewire or imaging catheter, or guide-catheter iatrogenic dissection [45, 46]. Thus, the use of this imaging should be limited to cases when the benefits outweigh the risks of the procedure.

### 4.3 Coronary Computed Tomography Angiography

While Coronary Computed Tomography Angiography (CCTA) provides an excellent evaluation of coronary and cardiac anatomy, definite diagnostic criteria have not been developed due to the lower spatial and temporal resolution than ICA [47]. Thus, a negative test cannot exclude the diagnosis of SCAD, as there is limited visualization of lumen and walls in the distal segments of small coronary arteries [48]. Tweet *et al*. [49] reported four primary coronary features in SCAD based on CCTA: (a) abrupt luminal stenosis (>50% diameter change over a length of 0.5 mm); (b) tapered luminal stenosis (>50% diameter change over a length of 5.0 mm); (c) intramural hematoma (IMH), which is thickening within the vessel wall and outside the true lumen corresponding to type 2 or 3; and (d) dissection flap, which gets visualized as linear hypodensity, such as in type 1 SCAD. Secondary high-risk factors that can be reported in SCAD are epicardial fat stranding, beading, coronary ectasia, and myocardial bridging [49, 50]. Additionally, coronary tortuosity was found to be a major anatomical risk in 78% of cases of SCAD by using the semiquantitative tortuosity scores [26]. Certain artifacts and diseases found in CCTA, such as coronary vasospasm, can mimic type 2 SCAD on ICA and acute coronary artery embolus, which can cause luminal occlusion and be mistaken as SCAD and need to be thoroughly assessed for an accurate diagnosis [51]. Nevertheless, to finalize the diagnostic criteria for utilizing CCTA in SCAD, larger studies need to be conducted.

### 4.4 Cardiac Magnetic Resonance (CMR)

CMR imaging is considered a diagnostic modality when SCAD has inconclusive ICA/CCTA results to confirm the presence of myocardial infarction and evaluate the degree of myocardial damage [52]. Characteristics of this mode of imaging have the following magnetic resonance imaging (MRI) findings in patients with SCAD: (a) late gadolinium enhancement, which could be transmural, affected by myocardium, subendocardial, and with patchy enhancement; (b) microvascular obstruction; and (c) intramural hematoma [52, 53]. A case-control study [54] reported the difference between SCAD and an ST-elevation myocardial infarction (STEMI) based on infarct size, where the former has a relatively small size on follow-up cardiac MR images, whereas another cohort [55] with 40 patients had two cases of SCAD reported due to the coupling of OCT with cardiac MRI. While currently, the diagnostic utility of CMR in SCAD is limited, there is significant potential for adopting CMR with 3D or T2 mapping in the future for coronary artery assessment [56].

### 4.5 Nuclear Myocardial Perfusion Imaging

Single photon emission computed tomography (SPECT) and Positron Emission Tomography (PET) are included in myocardial perfusion imaging (MPI) [51]. While this modality is used to evaluate perfusion during resting or stress conditions, in cases of atypical or stable chest pain, it can serve as an initial diagnostic test. If abnormalities are detected, this may prompt further evaluation with ICA/CCTA to assess for SCAD [2]. Another motive of utilizing MPI is to assess the extent and severity of involvement and opt for aggressive medical management with follow-up, while simultaneously avoiding invasive procedures, especially when a fixed defect (an unchanged perfusion defect at rest and stress) in single coronary arteries or non-viable myocardium is detected [51, 57]. Thus, nuclear myocardial perfusion imaging reports promising results for planning definitive treatment and long-term follow-up after the diagnosis of SCAD on angiography.

### 4.6 Echocardiography

Complementary evidence and support for the SCAD diagnosis can be provided through focused echocardiography or stress echocardiography (in patients with normal resting wall motion), which detects regional wall motion abnormalities due to myocardial ischemia or infarction [58]. Further, the formation of neo cavitation in the left ventricle as a sequela to SCAD-related IMH with acoustic characteristics of blood in its center can be detected by contrast echocardiography [59]. Such noninvasive imaging can also contribute to evaluating structural abnormalities and aortic root dimensions, which would help in the detection of genetic conditions and potentially reduce the risk of guide-catheter iatrogenic dissection [51].

### 4.7 Consensus for Diagnosis

Recent consensus statements from the American Heart Association (AHA, 2018) and the European Society of Cardiology (ESC, 2018) emphasize the importance of heightened clinical suspicion and precise interpretation of imaging modalities. Both recommend coronary angiography as the primary diagnostic method, careful use of intravascular imaging, and increasing reliance on noninvasive imaging (CCTA, CMR) for follow-up. These guidelines also advocate conservative treatment strategies to reduce the risk of complications inherent to invasive procedures, reflecting a substantial evolution in the approach to SCAD management over the last decade [39, 60].

### 4.8 Differential Diagnosis 

**Fig. 1.**
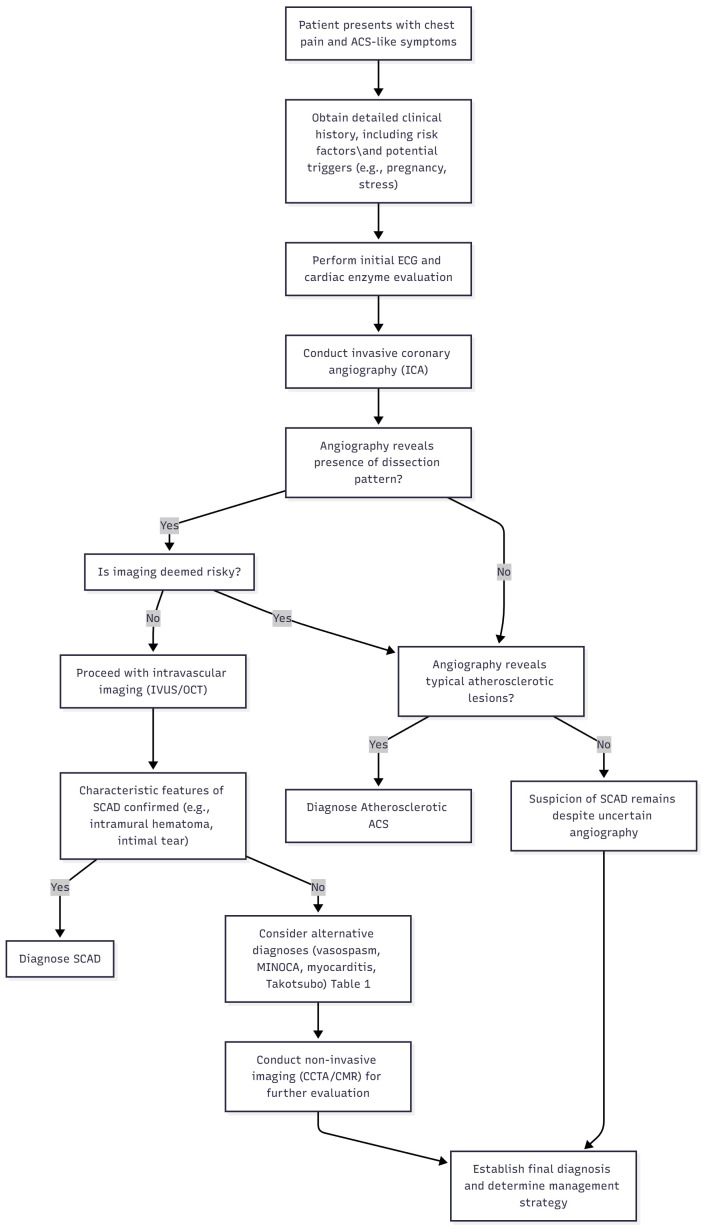
**Diagnostic algorithm with differentials to be considered**. ACS, Acute Coronary Syndrome; ECG, Echocardiogram; ICA, Invasive Coronary Angiogram; IVUS, Intravascular Ultrasound; OCT, Optical Coherence Tomography; SCAD, Spontaneous Coronary Artery Dissection; MINOCA, Myocardial Infarction with Non-obstructive Coronary Arteries; CCTA, Coronary Computed Tomography Angiography; CMR, Cardiac Magnetic Resonance.

The differential diagnosis of SCAD is complex. Clinicians must distinguish SCAD from a variety of conditions that can mimic its clinical and imaging features, such as atherosclerotic ACS, myocardial infarction with non-obstructive coronary arteries (MINOCA), coronary vasospasm, myocarditis, and even stress cardiomyopathy like Takotsubo syndrome (Fig. 1) (Table 1, Ref. [61, 62, 63, 64, 65, 66, 67, 68, 69, 70, 71, 72, 73, 74, 75, 76, 77, 78, 79, 80]) .

**Table 1.** **Differential diagnosis of spontaneous coronary artery dissection (SCAD)**.

Condition	Typical patient profile	Clinical presentation	Overlap with SCAD	Coronary imaging (ICA/IVUS/OCT/CCTA)	Cardiac imaging (Echo/CMR)	Response to therapy/clinical course	Key distinguishing features	References
Atherosclerotic ACS	Older patients (often 50s–70s), multiple CV risk factors (HTN, DM, smoking, hyperlipidemia)	Chest pain (predominant), ± dyspnea; ECG may show ST-elevation or non-ST changes	Chest pain, troponin rise, ECG changes	ICA: focal, eccentric stenosis, plaque rupture/thrombus; IVUS/OCT: calcified or lipid-rich plaque, rupture site	CMR: ischemic infarct patterns (subendocardial or transmural)	Requires PCI/revascularization; recurrence risk if untreated	Focal irregular plaques with rupture and thrombus vs diffuse long narrowing/intramural hematoma in SCAD	[61, 62, 63, 64, 65, 66, 67, 68]
MINOCA (including SCAD)	Often younger women with few/no traditional CV risk factors	Features of MI (elevated troponin, chest pain, ECG changes) without obstructive CAD	Chest pain, troponin rise, ECG changes	ICA: may show subtle SCAD (radiolucent flap, dual lumen, diffuse narrowing); IVUS/OCT may detect dissection plane	CMR: subendocardial infarct if ischemic; myocarditis can be excluded	Course depends on underlying cause; prognosis variable	SCAD is an important MINOCA cause; requires careful re-imaging to identify subtle dissection	[69, 70, 71, 72]
Coronary vasospasm	Often younger; association with smoking or stress	Episodic chest pain, often at rest or early morning; usually nitrate responsive	Chest pain, transient ECG changes, possible troponin rise	ICA: transient >90% narrowing, resolves with nitrates; spasm can be provoked with acetylcholine/ergonovine	Usually normal; edema/injury only if spasm prolonged	Responsive to nitrates and calcium channel blockers; recurrent but reversible	Functional, transient narrowing without structural wall disruption or intramural hematoma	[73, 74]
Takotsubo cardiomyopathy	Postmenopausal women; often triggered by emotional or physical stress	Chest pain, ECG ST-T changes, troponin rise	Chest pain, ECG changes, modest troponin rise	ICA: no culprit obstructive lesion; no dissection	Echo: apical ballooning with basal hyperkinesis (or variants); CMR: edema ± subtle non-ischemic LGE, no infarct-territory scar	Supportive therapy; LV function typically recovers within weeks	Characteristic apical ballooning pattern and transient LV dysfunction; absence of coronary dissection	[75, 76, 77, 78]
Myocarditis	Often younger; history of viral or inflammatory prodrome	Chest pain, fever, arrhythmias, troponin elevation	Chest pain, troponin rise, ECG changes	ICA: normal coronaries	CMR: edema and non-ischemic LGE (mid-wall/subepicardial) Echo: global or regional dysfunction	Supportive/anti-inflammatory treatment; variable but often self-limiting	Inflammatory myocardial injury pattern; no coronary dissection	[79, 80]

CV, cardiovascular; HTN, hypertension; DM, diabetes mellitus; MI, myocardial infarction; LGE, late gadolinium enhancement; PCI, percutaneous intervention; LV, left ventricle.

## 5. Management

Both the AHA [2] and ESC [37] guidelines emphasize the importance of conservative management and reserve revascularization strategies for select high-risk cases. The current goals of therapy are distinct from the strategies held for atherosclerotic acute coronary syndromes.

### 5.1 Conservative Approach

Due to the high rate of spontaneous healing evidenced by angiographic resolution, a conservative initial approach is advised for hemodynamically stable patients that do not show evidence of ischemia or high-risk anatomical features [81]. This includes intensive monitoring, antiplatelets, beta-blockers, risk-factor modification, and imaging on follow-up tailored as per every patient’s needs with scrutiny for recurrence or progression of the dissection [4, 82].

Dual Anti-Platelet Therapy (DAPT) with Aspirin and Clopidogrel is often the choice of drug for long-term secondary prevention, emphasized in the case of invasive management or previous history of myocardial infarction. Long-term beta blockade reduces arterial shear stress. Statins may be added in case of coexisting dyslipidemia. Both guidelines advise against the use of thrombolytics or anticoagulants due to the risk of propagation of dissection or clinical deterioration [81, 83].

SCAD-specific pharmacological interventions are lacking in literature and most of the above regimen are derived from ACS management. Both AHA and ESC acknowledged the lack of randomized control trials (RCT) to address the above [2, 37].

### 5.2 Revascularization Strategies 

Revascularization via percutaneous intervention (PCI) or coronary artery bypass grafting (CABG) is advised for select patients with evidence of ongoing ischemia, hemodynamic compromise, left main coronary artery involvement, or failure of the aforementioned conservative management.

PCI in SCAD is complicated by the fragile nature of the vessel wall, intramural hematomas, and ambiguous dissection planes. These increase the risks for technical failure because of iatrogenic extension of the dissection or hematoma propagation [84]. PCI also has long-term risks such as late stent thrombosis due to possible stent malpositioning post hematoma resorption [57]. Thus, PCI is sought only with the support of advanced imaging and if unavoidable. On the other hand, CABG can be preferred in patients with extensive multivessel SCAD, a previous failed PCI attempt, or left main dissection [4]. A meta-analysis by Martins *et al*. [83] analyzing 11 non-randomized studies revealed that patients undergoing revascularization as a first-line-of-intervention had an increased risk of recurrent SCAD in the same vessel vs. patients who underwent conservative management as first line. These findings underscore the importance of thoroughly identifying and communicating the potential risks before pursuing revascularization.

### 5.3 Pregnancy-Associated SCAD (p-SCAD)

SCAD that occurs during pregnancy or the early postpartum period is referred to as pregnancy-associated SCAD (p-SCAD). The estimated incidence is approximately 1.81 per 100,000 pregnancies, with the highest risk occurring in the first week postpartum [85]. Risk factors overlap with those for peripartum cardiomyopathy, including multiparity and hypertensive disorders of pregnancy, with additional associations noted with infertility treatments [85, 86].

The management of p-SCAD poses specific challenges. Due to the significant maternal and fetal risks, patients with p-SCAD are observed to undergo PCI interventions more often than non-pregnant SCAD patients, even though PCI demonstrates lower procedural success rates relative to conservative management. This discrepancy underscores the necessity for additional research into the specific intersection of imminent pregnancy management and SCAD outcomes [85].

A multidisciplinary approach is essential for conservative management, focusing on the well-being of both the mother and fetus during pregnancy, as well as lactational considerations postpartum. Just as in the management of ACS, low-dose aspirin and clopidogrel are typically regarded as safe and may be prescribed based on individual patient circumstances [87]. By contrast, pharmacologic options such as Angiotensin Converting Enzyme inhibitors (ACEi), Angiotensin Receptor Blockers (ARB), Angiotensin Receptor-Neprilysin Inhibitor (ARNi), Mineralocorticoid Receptor Antagonists (MRA), and Sodium-Glucose Cotransporter 2 (SGLT2) inhibitors are contraindicated during pregnancy. For breastfeeding mothers recovering from SCAD, ACEi, beta-blockers, and MRAs are regarded as acceptable, whereas ARBs, ARNi, and SGLT2 inhibitors lack sufficient safety evidence for recommendation [85, 86].

A critical factor in the management pathway is the utilization of imaging in cases of clinical progression despite conservative treatment. Coronary angiography, recognized as the gold standard for diagnosing SCAD, administers radiation doses that remain within established safety limits (approximately 20 mGy to the mother and 0.074 mGy to the fetus). Fetal radiation risks are typically regarded as negligible at doses below 50 mGy, with specific thresholds differing based on gestational age [87, 88].

### 5.4 Rehabilitation

Evidence-based protocols for cardiac rehabilitation (CR) for SCAD remain a conflicting field of literature. This privation puts patients who have been affected at high risk for recurrent events and associated psychological consequences [89]. Cardiac rehabilitation is a multidisciplinary secondary prevention intervention recommended after cardiac events, comprising psychosocial support, medical and cardiovascular risk factor modification, physical activity and education [90]. While CR is found to be associated with a decrease in morbidity and mortality in the cardiac population, certain obstacles impede its implementation [91, 92]. Binnie *et al*. [93] highlight areas that need further development, including a lack of awareness of SCAD among healthcare professionals, difficulty accessing the educational component during the recovery stage, a deficiency of tailored physical activity regimes according to individuals’ specific needs and physical capabilities, and the absence of psychosocial support with these programs.

To assess the effectiveness and safety of CR programs in SCAD, Chou *et al*. [89] designed the first prospective study, which additionally appraised components designed to meet the requirements of survivors. The most important factors focused on in the cohort were the recommended exercise threshold protocols aimed at decreasing arterial wall stress and promoting safe physical activities after SCAD, along with guidelines for addressing survivors’ psychosocial status, which has been underrepresented in past programs. With certain deductions, such as an upper limit of target heart rate aimed at 50–70% heart rate reserve, blood pressure maintained at a level no higher than 130/80, and frequent low-weight repetitions, the participants would be comfortable in improving their physical activity and reducing their fear of another attack [89]. The psychological burden post-SCAD, often characterized by anxiety and depression, highlights the need for CR programs to include comprehensive mental health support, as many patients become dependent on medication or behavioral therapy [94]. The study cohort of the SCAD Alliance [95] involving around 800 patients from multiple centers have shown significant associations of probable post-traumatic stress disorder diagnosis following a SCAD event, the prevalence being 34.7% for lifetime of the patient. While offering buddy and/or group-based opportunities for social contact, either in-person or online, has been proven to ameliorate psychosocial well-being and reduce depression scores, these programs still need to develop further to cater to the needs of female survivors where hurdles such as location and access, childcare, and costs exist [89, 96]. According to a census in the UK, 61% of females left the CR unattended due to the way these services were delivered [97]. While an alternative delivery method could be suggested, especially for physical activity, as most women are more likely to enroll in fitness classes rather than gym/circuit classes as suggested in referrals, SCAD recovery support programs should also start incorporating individualized telehealth or home-based support [98]. Thus, tailoring guidelines specifically for women can lead to an improvement in the prognosis for a major cohort of SCAD survivors.

Further large SCAD-CR cohorts and prospective program designs with a longer follow-up period are needed to assess the prevention of recurrent SCAD or major adverse cardiovascular events (MACE) events.

## 6. Conclusions

SCAD is an increasingly recognized cause of ACS, particularly in younger patients, females, and those without traditional cardiovascular risk factors. Despite advances in understanding its pathophysiology, diagnosis, and management, many challenges remain. SCAD is primarily associated with fibromuscular dysplasia, hormonal influences, and other predisposing conditions, emphasizing the need for tailored approaches in patient care.

Current diagnostic strategies rely heavily on coronary angiography, supplemented by intravascular imaging techniques such as optical coherence tomography and intravascular ultrasound for confirmation. Management of SCAD is generally conservative, prioritizing medical therapy and careful monitoring over revascularization, except in cases of ongoing ischemia or hemodynamic instability. Long-term follow-up and psychosocial support are critical due to the recurrent nature of SCAD and its profound impact on patients’ quality of life.

Future research should focus on refining diagnostic algorithms, identifying genetic and hormonal contributors, and optimizing individualized management strategies. Collaborative multicenter registries and studies are essential to further elucidate natural history, recurrence rates, and best practices for this complex condition. By advancing our knowledge and approach to SCAD, we can improve outcomes and enhance care for this unique patient population.
